# Supplementary material for: Development of a New Aggregation Method to Remove Nanoplastics from the Ocean: Proof of Concept Using Mussel Exposure Tests
Source: Biomimetics (Basel). 2024 May 18;9(5):303. doi: 10.3390/biomimetics9050303 (PMC11117817; doi:10.3390/biomimetics9050303)
Supplement: Supplementary file 1 [file biomimetics-09-00303-s001.zip › biomimetics-2998673-supplementary.pdf]

S1. Biochemical analysis

S1.1. Total protein content

The total protein content was recorded in each sample using the Bradford method [1]. This method entangles the formation of a complex between an acidic dye, Coomassie Blue G-250, and proteins in solution. This binding process shifts the dye color to an anionic blue form, which absorbs at 595 nm, thus allowing a spectrophotometric estimation of the protein concentration.

The Bradford reagent was prepared by melting 100 mg of blue Coomassie G-250 (BIO-RAD, *Hercules*, *California*, USA) in 100 mL of 100 mL of 85% phosphoric acid (Sigma-Aldrich, Darmstadt, Hesse, Germany) and 95% ethanol (Honeywell, Charlotte, NC, USA). The solution was diluted with ultrapure water (Milli-Q®) to the final volume of 1 L, filtered to eliminate precipitates, and stored at 4 ºC. A stock solution (4 mg/mL) of Bovine Serum Albumin (BSA; Nzytech, Lisboa, Lisboa, Portugal) was employed to obtain standards from 0 to 4 mg/mL by serial dilutions with PBS. The assay was achieved using a 96-well microplate (Greiner Bio-One GmbH, Kremsmünster, Kirchdorf an der Krems, Austria), and in each well, 20 μL of either BSA standard or sample and 180 μL of Bradford reagent were added. Both samples and standards were analyzed in duplicates. The absorbance was gauged at 595 nm using a microplate reader (BioRad Benchmark, San Francisco, California, USA). The BSA standards were used to build a calibration curve for the total protein concentration determination. The results were expressed in mg of total protein/mL.

S1.2. Superoxide dismutase activity (SOD)

The superoxide dismutase assay (EC 1.15.1.1) was based on the nitroblue tetrazolium (NBT) method, adapted from Sun *et al.*[2]. This method is based on the reduction of NBT to formazan. The NBT/NBT-formazan is mediated by superoxide radicals ($O_2^{\bullet-}$) formed by the reaction of xanthine with xanthine-oxidase (XOD) and can be measured spectrophotometrically at 560 nm. SOD contends with NBT for the dismutation of $O_2^{\bullet-}$, hindering its reduction. The inhibition level serves to measure SOD activity.

The assay was accomplished using a 96-well microplate (Greiner Bio-One GmbH, Kremsmünster, Kirchdorf an der Krems, Austria), adding to each well 10 μL of 3 mM EDTA (Riedel-Haën, Seelze, Lower Saxony, Germany), 200 μL of 50 mM PBS (pH 8.0) (Sigma-Aldrich, Darmstadt, Hesse, Germany), 10 μL of 3 mM $C_5H_4N_4O_2$ (Sigma-Aldrich, Darmstadt, Hesse, Germany), 10 μL of 0.75 mM NBT (Sigma-Aldrich, Darmstadt, Hesse, Germany) and 10 μL of SOD standard or sample.

The reaction was begun by adding 10 μL of 100 mU XOD (Sigma-Aldrich, Darmstadt, Hesse, Germany). Afterward, the absorbance was recorded at 560 nm every minute during 5 minutes using a plate reader (BioRad Benchmark, San Francisco, California, USA). Negative controls consisting of all components except SOD or sample were also included in the assay. Both negative controls and samples were analysed in duplicates. The results were expressed as the percentage of enzyme

inhibition. The SOD activity was normalized for the total protein content and expressed in percentage of inhibition per milligram of total protein (%inhibition/mg total protein).

S1.3. Catalase activity (CAT)

The catalase (CAT, EC 1.11.1.6) activity was determined according to a method described by Johansson and Borg and adapted for 96-well microplate[3]. This assay follows the reaction of CAT with methanol in the presence of H_2O_2 to obtain formaldehyde. Formaldehyde production is measured colorimetrically using Purpald as a chromogen. The compound formed upon the reaction of Purpald with formaldehyde is colorless; however, when oxidized, it turns to a purple color, which can be measured spectrophotometrically at 540 nm.

A stock solution of 4.25M of formaldehyde (Sigma, Darmstadt, Hesse, Germany) was used to prepare standards in buffer containing 25mM of potassium phosphate (Sigma-Aldrich, Darmstadt, Hesse, Germany, pH 7.0), 1 mM of EDTA (Riedel-de Haën, Seelze, Lower Saxony, Germany) and 0.1% of BSA (Nzytech, Lisboa, Portugal), with a range of formaldehyde concentrations from 0 to 75 μM.

The assay was completed using a 96-well microplate (Greiner Bio-One GmbH, Kremsmünster, Kirchdorf an der Krems, Austria), and in each well, 20 μL of standards or samples, 100 μL of 100 mM potassium phosphate (Sigma-Aldrich, Seelze, Lower Saxony, Germany; pH 7.0), and 30 μL of methanol were added to each well. The reaction was begun by adding 20μL of 0.035M H_2O_2 (Sigma-Aldrich, Seelze, Lower Saxony, Germany). The microplate was incubated in the dark for 20 minutes at room temperature. Subsequently, 30μL of 10M of potassium hydroxide (Chem-Lab, Zedelgem, Belgium) were added to stop the reaction, and 30μL of Purpald (34.2 mM in 0.5 M of HCl, Aldrich, Seelze, Lower Saxony, Germany) were added to each well. Microplates incubated in the dark for 10 minutes at room temperature. A final incubation of 5 minutes under the same conditions was carried out after adding 10μL of KIO_4 (65.2 mM in 0.5 M of KOH; Chem-Lab, Zedelgem, Belgium). The absorbance was recorded in a microplate reader (Synergy HTX, BioTek, Winooski, VT, USA) at 540 nm. Both samples and standards were analyzed in duplicates.

Catalase activity was calculated based on the assumption that one unit of catalase will form 1.0 nmol of formaldehyde/minute at 25 ºC. The formaldehyde standards were used to build a calibration curve for the total enzyme activity determination. Results were expressed concerning the total protein concentration (nmol/min/mg total protein).

S1.4. Glutathione peroxidase activity (GPX)

Glutathione peroxidase activity (GPx, EC 1.11.1.9) was determined following the procedure described by Lawrence and Burk [4] and adapted for a 96-well microplate (Greiner Bio-One GmbH, Kremsmünster, Kirchdorf an der Krems, Austria). This assay determines the GPx activity through an indirect measurement based on the oxidation of NADPH to $NADP^+$, which can be assessed spectrophotometrically at 340 nm.

In this assay, 120 µL of buffer composed of 50 mM of potassium phosphate (Sigma-Aldrich, Seelze, Lower Saxony, Germany; pH 7.3) and 5mM of EDTA (Riedel-de Haën, Seelze, Lower Saxony, Germany) and 50 µL of the co-substrate mixture comprised of 4 mM N_3Na (Sigma-Aldrich, Seelze, Lower Saxony, Germany), 1 mM NADPH (Sigma-Aldrich, Seelze, Lower Saxony, Germany), 4 U/mL of glutathione reductase (GSST-reductase; Sigma, Seelze, Lower Saxony, Germany) and 4 mM of reduced glutathione (GSH; Sigma, Seelze, Lower Saxony, Germany) and 20 µL of each sample was added to microplate wells. The reaction was then initiated, adding 20 µL of 15 mM of cumene hydroperoxide (Sigma-Aldrich, Seelze, Lower Saxony, Germany). The absorbance was recorded every minute at 340 nm during 6 minutes using a microplate reader (Synergy HTX, BioTek, Winooski, VT, USA). The reaction rate was measured using the β-NADPH extinction coefficient of 3.73 mM^{-1} cm^{-1} and the GPx activity. Results were expressed related to the total protein concentration (nmol/min/mg total protein).

S1.5. Total antioxidant Capacity (TAC)

The Total Antioxidant Capacity (TAC) was determined following the method described by Kambayashi *et al*. [5], based on the colorimetric detection of ABTS, a blue-green chromophore that decreases its intensity in the presence of antioxidants. In this assay, Trolox, a compound analog to vitamin E, is equivalent to antioxidant capacity.

A stock solution of 1.5M of Trolox (Sigma, Seelze, Lower Saxony, Germany) was used to prepare standards in 50 mM phosphate buffer (Sigma-Aldrich, Seelze, Lower Saxony, Germany, pH 7.3) with a range of Trolox concentrations from 0 to 0.33 mM.

Briefly, 10 µL of the sample, 10 µL of 90 µM of myoglobin (Sigma, Seelze, Lower Saxony, Germany), 150 µL of 600µM of ABTS (2,2′-Azino-bis(3-ethylbenzothiazoline-6-sulfonic acid); (Alfa Aesar, Karlsruhe, Karlsruhe, Germany) were added to a 96-well microplate (Greiner Bio-One GmbH, Kremsmünster, Kirchdorf an der Krems, Austria). After initiating the reaction, a 5-minute incubation at room temperature was carried out by adding 40 µL of 250 µM of hydrogen peroxide (Sigma-Aldrich, Seelze, Lower Saxony, Germany). The absorbance was read employing a microplate reader (Synergy HTX, BioTek, Winooski, VT, USA) at 415 nm. Both samples and standards were analysed in duplicate. Trolox standards were used to obtain a calibration curve to determine total antioxidant capacity. Results were expressed related to the total protein concentration (mmol/ mg total protein).

S1.6. Glutathione s-transferase activity (GST)

Glutathione S-Transferase activity (GST, EC 2.5.1.18) was measured following the method reported by Habig *et al.* [6] and adapted for a 96-well microplate. This method measures the increase in the absorbance at 340 nm that follows the formation of a conjugate between glutathione (GSH) and l-chloro-2,4-dinitrobenzene (CDNB).

To perform this assay, 20 µL of the sample and 180 µL of 200 mM reduced glutathione (GSH; Sigma-Aldrich, Seelze, Lower Saxony,

Germany) and 100mM of CDNB (Sigma-Aldrich, Seelze, Lower Saxony, Germany) in Dulbecco's phosphate saline buffer were added to the 96-well microplate (Greiner Bio-One GmbH, Kremsmünster, Kirchdorf an der Krems, Austria).

The enzyme activity was determined by recording the absorbance every minute at 340 nm for 6 minutes through a microplate reader (Synergy HTX, BioTek, Winooski, VT, USA). Changes in absorbance per minute were estimated, and the reaction rate was calculated using the CDNB extinction coefficient of 5.3 mM^{-1} cm^{-1}. Results were expressed related to the total protein concentration (nmol/min/mg total protein).

S1.7. Lipid peroxidation (MDA content)

Lipid peroxidation was conducted following the thiobarbituric acid reactive species (TBARS) method described by Ohkawa *et al.*[7] and modified by Madeira *et al.*[8]. TBARS method follows the reaction of thiobarbituric acid (TBA) with malondialdehyde bis(dimethyl acetal) (MDA) to produce a final colorimetric compound that absorbs at 530 nm and which is proportional to MDA content.

A stock solution of 1 µM of MDA (Merck Millipore, Burlington, Massachusetts, USA) was used to prepare TBARS standards in Milli-Q® water with MDA concentrations ranging from 0 to 0.1 µM.

The assay was performed by adding 5 µL of each sample or standard and 45 µL of 50 mM of monobasic sodium phosphate buffer (Sigma-Aldrich, Seelze, Lower Saxony, Germany) to individual microtubes (1.5 mL). Then, 12.5 µL of sodium dodecyl sulfate (8.1 % SDS, Merck, Darmstadt, Hesse, Germany), 93.5 µL of trichloroacetic acid (TCA 20 %, pH 3.5; Panreac, Barcelona, Cataluña, Spain), 93.5 µL of thiobarbituric acid (TBA 1 %, Sigma-Aldrich, Seelze, Lower Saxony, Germany) and 50.5 93.5 µL of Milli-Q® water were added to each microtube. This mixture was then centrifuged at 2,000xg for 1 minute; subsequently, the lids were punctured with a needle to incubate in boiling water (10 minutes at 100ºC). Afterward, the microtubes were placed on ice and allowed to cold, then 62.5 µL of Milli-Q® water was added into each microtube and centrifuged at 2,000 x g for 1 minute. Finally, duplicates of 150 µL of the supernatants of each sample or standard were added into a 96-well microplate (Greiner Bio-One GmbH, Kremsmünster, Kirchdorf an der Krems, Austria), and the absorbance was read at 530 nm employing a microplate reader (Synergy HTX, BioTek, Winooski, VT, USA).

MDA was used as the standard to build a calibration curve. Results were expressed related to total protein concentration (nmol/ mg total protein).

S1.8. Total Ubiquitin Content (Ub)

Total ubiquitin was quantified through an indirect Enzyme-linked Immunosorbent Assay (ELISA) described by Njemini *et al.*[9], and adapted for a 96-well microplate, as described by Rosa *et al.*[10].

A stock solution of ubiquitin (0.8 µg/mL, UBPBio, Dallas, TX, USA) was used to prepare ubiquitin standards in PBS from 0 to 0.8 µg/mL.

Briefly, 50μL of the sample or standards were added into the 96-well microplate (Greiner Bio-One GmbH, Kremsmünster, Kirchdorf an der Krems, Austria) and incubated overnight at 4ºC. Then, the microplates were washed (3 times) with a phosphate-buffered saline solution (PBS; 140 mM of NaCl, Panreac, Barcelona, Cataluña, Spain); 10 mM of Na_2HPO_4, (Sigma-Aldrich, St. Louis, MO USA); 3 mM of KCl, (Merck, Darmstadt, Hesse, Germany; pH 7.3) containing 0.05% of Tween-20 solution (Sigma-Aldrich, Seelze, Lower Saxony, Germany) and 200 μL of blocking solution (PBS with 1% of BSA) (Nzytech, Lisboa, Portugal) was added to each microplate well.

After 90 minutes of incubation at 37ºC (Labnet, Edison, NJ, USA), microplates were rewashed (3x with PBS with 0.05% of Tween-20). Afterward, 50μL of the primary antibody (Ub (P4D1) Sc-8017, mouse monoclonal IgG, Santa Cruz Biotechnology, Portugal; diluted to 0.5 μg/mL in PBS with 1% BSA) were added to each well of the 96-well microplate and incubated overnight at 4 ºC. Subsequently, another washing step was carried out, followed by adding 50 μL of secondary antibody (anti-mouse IgG Fc specific alkaline phosphatase, Sigma-Aldrich, Seelze, Lower Saxony, Germany; diluted to 1.0 μg/mL in PBS with 1% BSA) was added to each microplate well and incubated for 90 minutes at 37 ºC. Another washing step was performed, and once completed, 100μL of alkaline-phosphatase substrate (100 mM of NaCl (Panreac, Barcelona, Cataluña, Spain), 100 mM of Tris-HCl (Sigma-Aldrich, Seelze, Lower Saxony, Germany), 50 mM of $MgCl_2$ (Sigma-Aldrich, Seelze, Lower Saxony, Germany) and 27 mM of PnPP (4-nitrophenyl phosphate disodium salt hexahydrate, pH 8.5; Sigma-Aldrich, Seelze, Lower Saxony, Germany), was added to each microplate well. Then, microplates were incubated for 30 min at room temperature was carried out before adding 50 μL of stop solution (3M NaOH, Panreac, Barcelona, Cataluña, Spain) was added to each microplate well. The absorbance was registered at 405 nm using a microplate reader (Synergy HTX, BioTek, Winooski, VT, USA).

Ubiquitin standards were used to build a calibration curve to determine the total ubiquitin content. Results were expressed related to total protein concentration (μg/ mg total protein).

Table S1. DLS measurements of MPs in FMQ in different concentrations of FIL and chitosan. Data are presented as (mean ± sd).

MPs (mg/mL)	FIL (mg/mL)	Chitosan (μg/mL)	Z-Average (nm)	PDI	ζ-Pot (mV)	E. M. (cm^2/Vs) [1]
0.0200	-	-	1121.90 ± 62.11	0.20 ± 0.17	-3.9 ± 1.44	-0.00003 ± 0.000011
0.0190	69.22	-	1166.81 ± 142.30	0.33 ± 0.14	-15.2 ± 1.68	-0.000118 ± 0.000013
0.0182	132.15	-	1026.99 ± 78.45	0.35 ± 0.15	-7.37 ± 2.61	-0.000057 ± 0.00002
0.0196	-	0.006	1259.78 ± 137.98	0.42 ± 0.29	-11.53 ± 1.82	-0.000090 ± 0.000014
0.0186	-	0.021	1158.57 ± 96.73	0.36 ± 0.23	-16.65 ± 1.36	-0.000129 ± 0.000010
0.0182	-	0.027	1076.93 ± 163.61	0.16 ± 0.02	-10.31 ± 1.36	-0.000080 ± 0.000010

0.0179	129.79	0.005	1134.92 ± 67.00	0.32 ± 0.18	-7.21 ± 0.96	-0.000057 ± 0.000020
0.0170	123.71	0.019	1146.30 ± 53.91	0.18 ± 0.15	-5.74 ± 1.76	-0.000045 ± 0.000014

[1] E. M.: electrophoretic mobility.

The data presented refers only to the heteroaggregation tests of MPs in Milli-Q® water since the results were not conclusive or relevant to be applied in synthetic seawater.

S2. Microplastics

S2.1. Catalase activity (CAT)

The results from CAT activity measured in the gills and digestive glands of *M. galloprovincialis* are represented in Figure S1.

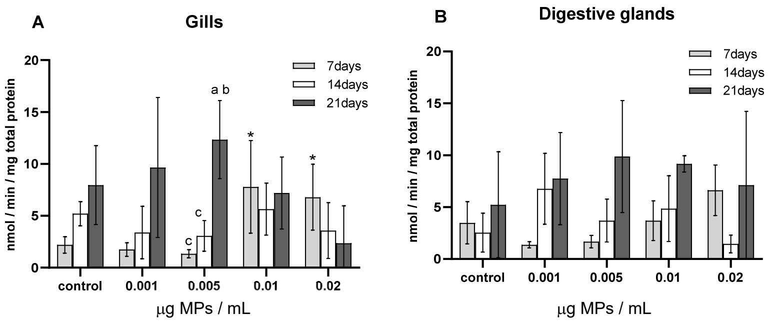

Figure S1. Catalase activity (mean ± sd) in *M. galloprovincialis* exposed to different concentrations of MPs measured in gills (A) and digestive glands (B) after 7, 14, and 21 days. Significant differences ($p<0.05$) compared to controls are denoted by *. Legend: lowercase letters a, b, and c indicate significant differences ($p<0.05$) between 7, 14, and 21 days of exposure, respectively.

Before the bioassays began (T0), CAT activity was measured, showing values of 1.23 ± 1.16 nmol/min/mg total protein for the gills and 2.92 ± 2.40 nmol/min/mg total protein for the digestive glands. However, the statistical analysis displayed no significant differences between the controls and the exposed mussels, except for the mussels' gills, after 14 days of exposure to MPs ($p=0.025$).

Catalase showed its highest activity value (12.35 ± 3.76 nmol/min/mg total protein) in the gills after 21 days of exposure to 0.005 µg MPs/mL. In the digestive glands, the highest value (9.89 ± 5.39 nmol/min/mg total protein) was measured at the same MPs concentration (0.005 µg MPs/mL) and exposure period (21 days).

Regarding the gills, catalase activity remained constant after 7 and 14 days of exposure of 0.001 µg MPs/mL and 0.005 µg MPs/mL compared to controls. In contrast, in the highest MPs concentrations (0.01 and 0.02 µg MPs/mL) after 7 days, CAT activities were significantly higher than the controls ($p=0.039$ and $p=0.016$, respectively). Regarding the 0.005 µg MPs/mL bioassay, catalase activity showed an evident increase after 21 days of exposure to MPs compared to 7 days and 14 days values ($p=0.006$ and $p= 0.014$, respectively).

Concerning digestive glands, no significant differences in CAT activity ($p>0.05$) were found between exposed mussels and controls.

Nonetheless, the highest CAT activities were found after 21 days of exposure.

In general, after 21 days of exposure to MPs, catalase activity increased according to MPs tested concentrations, up to 0.005 µg MPs/mL, followed by a decrease.

S2.2. Glutathione-S-transferase (GST)

GST activity was determined in the gills and digestive glands of *M. galloprovincialis,* and the results are shown in Figure S2.

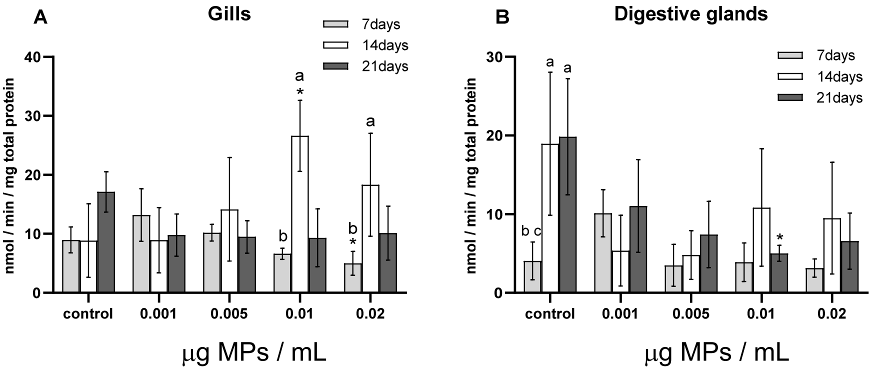

Figure S2. GST activity (mean ± sd) in *M. galloprovincialis* exposed to different MPs concentrations measured in gills (A) and digestive glands (B) after 7, 14, and 21 days of exposure. Significant differences ($p<0.05$) compared to controls are denoted by *. Legend: lowercase letters a, b, and c indicate significant differences ($p<0.05$) between 7, 14, and 21 days of exposure, respectively.

Before the bioassays (T0) began, GST activity was measured, showing values of 6.18 ± 4.02 nmol/min/mg total protein in the gills and 4.49 ± 2.27 nmol/min/mg total protein in the digestive glands. No significant differences were obtained compared to controls, except for mussels' gills after 21 days of exposure ($p=0.021$) and digestive glands after 14 and 21 days of exposure to MPs ($p=0.021$ and $p=0.014$, respectively).

GST activities in gills ranged from 4.99 ± 2.03 nmol/min/mg total protein (mussels exposed to 0.02 µg/mL MPs for 7 days) to 26.61 ± 6.01 nmol/min/mg total protein (after 14 days of exposure to 0.01 µg/mL MPs). After 14 days of exposure, the highest GST activities were registered in mussels exposed to 0.005, 0.01, and 0.02 µg/mL MPs compared to controls; however, only the mussels exposed to 0.01 µg/mL of MPs showed significant difference compared to controls.

The highest GST activity was measured in the digestive glands of control mussels after 21 days of exposure (19.84 ± 7.39 nmol/min/mg), while the lowest value was 3.15 ± 1.16 nmol/min/mg total protein, determined in the digestive glands of mussels exposed to 0.02 µg/mL MPs for 7 days.

S2.3. Total antioxidant capacity (TAC)

TAC was measured in the gills and digestive glands of *M. galloprovincialis*, and the results are presented in Figure S3.

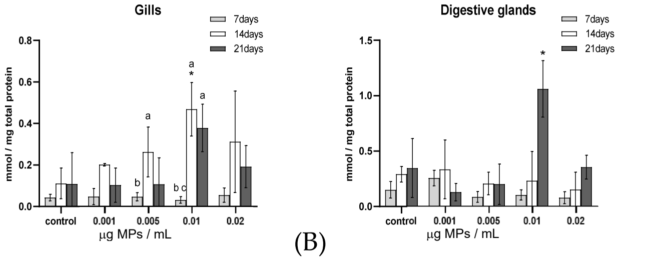

(A) (B)

Figure S3. Total antioxidant capacity (mean ± sd) in *M. galloprovincialis* exposed to different MPs concentrations measured in gills (A) and digestive glands (B) after 7, 14, and 21 days. Significant differences (p<0.05) compared to controls are denoted by *. Legend: lowercase letters a, b, and c indicate significant differences (p<0.05) between 7, 14, and 21 days of exposure times, respectively.

No results were obtained before the beginning of the experiment (T0). TAC values in the gills of treated mussels after 14 and 21 days of exposure show a trend to increase according to MPs tested concentrations. The highest TAC value of 0.47 ± 0.13 nmol/min/mg total protein was measured after 14 days of exposure to 0.01 µg/mL MPs, significantly different (p=0.025) from controls.

Concerning digestive glands, a significant increase was found (1.06 ± 0.25 nmol/min/mg total protein, p=0.025) in mussels exposed for 21 days to 0.01 µg/mL of MPs. No significant differences (p>0.05) were obtained for the other concentrations and exposure periods.

S2.4. Lipoperoxidation (MDA content)

Lipoperoxidation was measured in the gills and digestive glands of *M. galloprovincialis. T*he results, expressed in terms of MDA concentration, are presented in Figure S4.

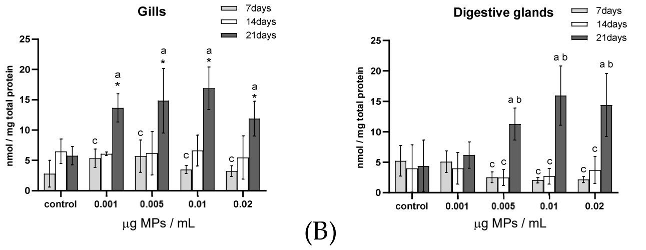

(A) (B)

Figure S4. MDA concentration (mean ± sd) in *M. galloprovincialis* exposed to different MPs concentrations measured in gills (A) and digestive glands (B) after 7, 14, and 21 days of exposure. Significant differences (p<0.05) compared to controls are denoted by *. Legend: lowercase letters a, b, and c indicate significant differences (p<0.05) between 7, 14, and 21 days of exposure, respectively**.**

Before the bioassays began (T0), lipid peroxidation (LPO) was measured, presenting MDA concentration values of 5.7 ± 2.6 nmol/min/mg total protein in the gills and 4.26 ± 3.0 nmol/min/mg total protein for the digestive glands. No significant differences were found between the controls.

Results revealed that after 21 days of exposure, the MDA concentration in the gills significantly increased (p<0.05) at all exposure

concentrations, reaching the highest value (16.91 nmol/min/mg total protein) at 0.01 μg /mL of MPs. Although there were no significant differences regarding the controls in the digestive glands, the same trend was registered.

S2.5. Total ubiquitin (Ub)

Total ubiquitin concentration was measured in the gills and digestive glands of *M. galloprovincialis*, and the results are represented in Figure S5.

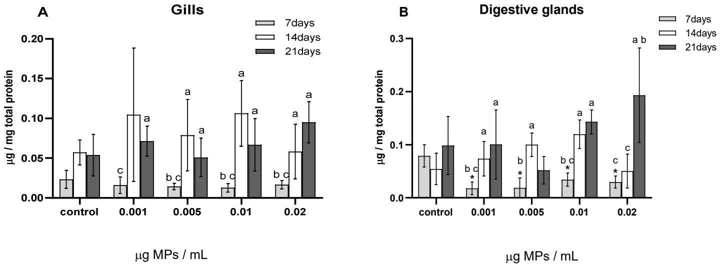

Figure S5. Total ubiquitin (mean ± sd) in *M. galloprovincialis* exposed to different MPs concentrations measured in gills (A) and digestive glands (B) after 7, 14, and 21 days of exposure. Significant differences ($p<0.05$) compared to controls are denoted by *. Legend: lowercase letters a, b, and c indicate significant differences ($p<0.05$) between 7, 14, and 21 days of exposure, respectively**.**

Before the beginning of the bioassays (T0), total ubiquitin was measured and presented an average concentration of 0.014 ± 0.005 nmol/min/mg total protein in the gills and 0.056 ± 0.052 nmol/min/mg total protein in the digestive glands which. Significant differences ($p<0.05$) were found between the controls, except in the gills of mussels exposed to MPs after 7 days of exposure.

Despite the differences within the exposure periods, the total ubiquitin content did not show significant differences in the gills of mussels exposed to MPs regardless of the concentration applied comparatively to controls. There was a trend ($p>0.05$) in the digestive glands of ubiquitin concentrations to increase according to the concentration and exposure period.

In general, the total ubiquitin values were higher in the digestive glands compared to the gills.

Discussion

CAT activity, regardless of the tissue, in mussels exposed to lower MPs concentrations (0.001 and 0.005 μg MPs/mL) from 7 to 14 days of exposure remained constant compared to controls, whilst after 21 days of exposure, a trend to increase was found, suggesting that, in these concentrations, MPs may induce a stress response after long-term exposure. On the other hand, in the highest concentrations (0.01 and 0.02 μg MPs/mL) after 7 days of exposure, a noteworthy increase in CAT activity was observed in mussels' gills (Figure 3.10A), which was also reported by Ribeiro *et al.* [11] in the gills of *S. plana* after three days of exposure to MPs. However, the concentrations applied in that study were much higher (1 mg/L). In contrast, no significant differences were found in the digestive glands, suggesting that MPs did not induce

substantial oxidative stress to trigger the activation of compensatory mechanisms by CAT in this tissue.

GST activity remained constant in both tissues' lower MPs exposure concentrations (0.001 and 0.005 μg MPs/mL). Avio *et al.* [12] found similar results in the digestive glands of *M. galloprovincialis* after 7 days of exposure to PS MPs. However, in the gills, a trend to increase in GST activity was found in the highest exposure concentrations (0.01 and 0.02 μg MPs/mL) after 14 days of exposure, which is in agreement with previous findings in studies carried out with *S.plana* exposed to PS MPs [11]. In the digestive glands, it is not possible to infer any significant changes in GST activities between controls and exposed mussels after 14 and 21 days of exposure. Nevertheless, it must be noted that significant differences were found between controls and T0. These differences may be attributed to additional stress induced during the manipulation and preparation of mussels for the bioassay or due to age, sex, and stage of sexual maturity differences between the animals [13],[14].

Concerning the mussel's gills, despite the increasing trend, TAC did not show significant differences for all tested MPs concentrations and exposure times, except for 0.01 μg MPs/mL after 14 days of exposure. At the same concentration of MPs in the digestive glands, TAC increased significantly after 21 days compared to controls. Nevertheless, in all the other MPs concentrations and exposure periods, TAC in the digestive glands remained constant compared to controls. Accordingly, Avio *et al.* [12] reported limited effects on the total antioxidant capacity of *M. galloprovincialis* after 7 days of exposure to PS MPs. Moreover, Pittura *et al.* [15] also showed no significant fluctuations in the total antioxidant capacity in the digestive glands of *M. galloprovincialis* after 7, 14, and 28 days of exposure to low-density polyethylene microparticles.

The results revealed that, for all tested concentrations of MPs, in mussel's gills, there were no significant differences in lipoperoxidation levels from 7 to 14 days of exposure, however, a substantial increase was found after 21 days of exposure compared to controls. The same trend was observed in the digestive glands, with LPO remaining constant at the beginning, followed by a trend to increase after 21 days of exposure compared to controls. Studies from Gonçalves *et al.* showed no alterations in the lipid peroxidation levels in the gills and digestive glands of *M. galloprovincialis* after 2 days of exposure to PS MPs [16]. Nevertheless, LPO gill's results suggest that after 21 days of exposure to MPs, mussels could not develop an effective antioxidant mechanism to counteract the increase of ROS, consequently causing cellular damage with the production of lipid peroxides [17],[18].

Indeed, for the total ubiquitin concentration, the results showed a significant increase between 7 and 21 days of exposure for all concentrations of MPs tested in the gills or digestive gland. Although this increase was not significant compared to the controls, it is worth mentioning that the Ub values in the controls were significantly higher than the T0 values. Thus, the results suggest that exposure to MPs

induced protein damage, consequently leading to ubiquitin production.

Regarding MPs exposure tests, during the 21 days of exposure it was shown that the tested concentrations were not lethal for *M. galloprovinciallis,* which was expected, considering that Galloway *et al.* [19] reported that the uptake of MPs rarely causes death. Conversely, mussel mortality was observed in the trial with higher concentrations of NPs. Although, considering that at this concentration, overall, no significant changes occurred in terms of antioxidant enzyme activities, it is unlikely that oxidative stress was the leading cause of the reported deaths.

References

1. Bradford, M.M. A Rapid and Sensitive Method for the Quantification of Microgram Quantities of Protein Utilizing the Principle of Protein-Dye Binding. *Anal. Biochem.* **1976**, *72*, 248–254. https://doi.org/10.1016/0003-2697(76)90527-3.
2. Sun, Y.; Oberley, L.W.; Li, Y. A Simple Method for Clinical Assay of Superoxide Dismutase. *Clin. Chem.* **1988**, *34*, 497–500. https://doi.org/10.1093/clinchem/34.3.497.
3. Johansson, L.H.; Håkan Borg, L.A. A Spectrophotometric Method for Determination of Catalase Activity in Small Tissue Sam-ples. *Anal. Biochem.* **1988**, *174*, 331–336. https://doi.org/10.1016/0003-2697(88)90554-4.
4. Lawrence, A.; Burk, R.F. Glutathione Peroxidase Activity in Selenium-Deficient Rat Liver. Biochem. *Biophys. Res. Commun.* **1976**, *71*, 952–958. https://doi.org/10.1016/0006-291X(76)90747-6.
5. Nutr, J.C.B.; Kambayashi, Y.; Binh, N.T.; Asakura, H.W.; Hibino, Y. Efficient Assay for Total Antioxidant Capacity in Human Plasma Using a 96-Well Microplate. *J. Clin. Biochem. Nutr.* **2009**, *44*, 46–51.
6. Habig, W.H.; Pabst, M.J.; Jakoby, W.B. Glutathione S-Transferases—The First Enzymatic Step in Mercapturic Acid Formation. *J. Biol. Chem. Phys.* **1974**, *249*, 7130–7140.
7. Ohkawa, H.; Ohishi, N.; Yagi, K. Assay for Lipid Peroxides in Animal Tissues by Thiobarbituric Acid Reaction. *Anal. Biochem.* **1979**, *95*, 351–358. https://doi.org/10.1016/0003-2697(79)90738-3.
8. Madeira, D.; Costa, P.M.; Vinagre, C.; Diniz, M.S. When Warming Hits Harder: Survival, Cellular Stress and Thermal Limits of Sparus Aurata Larvae under Global Change. *Mar. Biol.* **2016**, *163*, 91. https://doi.org/10.1007/s00227-016-2856-4.
9. Njemini, R.; Demanet, C.; Mets, T. Comparison of Two ELISAs for the Determination of Hsp70 in Serum. *J. Immunol. Methods* **2005**, *306*, 176–182. https://doi.org/10.1016/j.jim.2005.08.012.
10. Rosa, R.; Trubenbach, K.; Pimentel, M.S.; Boavida-Portugal, J.; Faleiro, F.; Baptista, M.; Dionisio, G.; Calado, R.; Portner, H.O.; Repolho, T. Differential Impacts of Ocean Acidification and Warming on Winter and Summer Progeny of a Coastal Squid (*Loligo vulgaris*). *J. Exp. Biol.* **2014**, *217*, 518–525. https://doi.org/10.1242/jeb.096081.
11. Ribeiro, F.; Garcia, A.R.; Pereira, B.P.; Fonseca, M.; Mestre, N.C.; Fonseca, T.G.; Ilharco, L.M.; Bebianno, M.J. Microplastics Effects in Scrobicularia Plana. *Mar. Pollut. Bull.* **2017**, *122*, 379–391. https://doi.org/10.1016/j.marpolbul.2017.06.078.
12. Avio, C.G.; Gorbi, S.; Milan, M.; Benedetti, M.; Fattorini, D.; D'Errico, G.; Pauletto, M.; Bargelloni, L.; Regoli, F. Pollutants Bioavailability and Toxicological Risk from Microplastics to Marine Mussels. *Environ. Pollut.* **2015**, *198*, 211–222. https://doi.org/10.1016/j.envpol.2014.12.021.

13. Box, A.; Sureda, A.; Galgani, F.; Pons, A.; Deudero, S. Assessment of Environmental Pollution at Balearic Islands Applying Oxidative Stress Biomarkers in the Mussel *Mytilus galloprovincialis*. *Comp. Biochem. Physiol. C Toxicol. Pharmacol.* **2007**, *146*, 531–539. https://doi.org/10.1016/j.cbpc.2007.06.006.
14. Cole, M.; Liddle, C.; Consolandi, G.; Drago, C.; Hird, C.; Lindeque, P.K.; Galloway, T.S. Microplastics, Microfibres and Nanoplastics Cause Variable Sub-Lethal Responses in Mussels (*Mytilus* spp.). *Mar. Pollut. Bull.* **2020**, *160*, 111552. https://doi.org/10.1016/j.marpolbul.2020.111552.
15. Pittura, L.; Avio, C.G.; Giuliani, M.E.; d'Errico, G.; Keiter, S.H.; Cormier, B.; Gorbi, S.; Regoli, F. Microplastics as Vehicles of Environmental PAHs to Marine Organisms: Combined Chemical and Physical Hazards to the Mediterranean Mussels, *Mytilus galloprovincialis*. *Front. Mar. Sci.* **2018**, *5*, 103. https://doi.org/10.3389/fmars.2018.00103.
16. Martins, M.; Sobral, P.; Costa, P.M.; Costa, M.H. An Assessment of the Ability to Ingest and Excrete Microplastics by Fi Lter-Feeders : A Case Study with the Mediterranean Mussel. *Environ. Pollut.* **2019**, *245*, 600–606. https://doi.org/10.1016/j.envpol.2018.11.038.
17. de Zwart, L.L.; Meerman, J.H.; Commandeur, J.N.; Vermeulen, N.P. Biomarkers of Free Radical Damage. *Free. Radic. Biol. Med.* **1999**, *26*, 202–226. https://doi.org/10.1016/s0891-5849(98)00196-8.
18. Lesser, M.P. Oxidative Stress in Marine Environments : Biochemistry and Physiological Ecology. *Annu. Rev. Physiol.* **2006**, *68*, 253–278. https://doi.org/10.1146/annurev.physiol.68.040104.110001.
19. Galloway, T.S.; Cole, M.; Lewis, C. Interactions of Microplastic Debris throughout the Marine Ecosystem. *Nat. Ecol. Evol.* **2017**, *1*, 0116. https://doi.org/10.1038/s41559-017-0116.
